# Teprotumumab’s Impact on Proptosis in Long-duration Thyroid Eye Disease: A Systematic Review and Meta-analysis

**DOI:** 10.17925/EE.2024.20.2.13

**Published:** 2024-10-15

**Authors:** Nicholas A Householder, Coby Ray

**Affiliations:** 1. School of Medicine, Lubbock Campus, Texas Tech University Health Sciences Center, Lubbock, TX, USA; 2. Ophthalmology, School of Medicine, Lubbock Campus, Texas Tech University Health Sciences Center, Lubbock, TX, USA

**Keywords:** Exophthalmos, Graves’ disease, Graves’ ophthalmopathy, Graves’ orbitopathy, proptosis, teprotumumab, thyroid eye disease

## Abstract

**Background:** Long-duration thyroid eye disease (TED) may present with persistent proptosis despite the absence of inflammatory symptoms, and treatment options have been limited to surgical intervention. Recently, teprotumumab, a monoclonal antibody, has garnered interest as a non-surgical option to reduce proptosis in such cases. This systematic review investigates the impact of teprotumumab on reducing proptosis in long-duration TED. **Methods:** A search was conducted across major online databases, and data were aggregated from observational studies, clinical trials and case series. Nine studies met the inclusion criteria. Cumulative and weighted effect measures were synthesized. The biases and limitations of each study were assessed. **Results:** Existing evidence shows teprotumumab to be highly efficacious in reducing proptosis in chronic TED; however, there are significant limitations in the quality of existing evidence. The cumulative meta-analysis reveals a mean proptosis reduction of 3.05 ± 0.54 mm across 182 orbits from nine studies, and the weighted meta-analysis shows a mean reduction of 2.69 ± 0.53 mm across 172 orbits from eight studies. **Discussion:** While existing clinical studies are open to bias and intrinsically limited, the meta-analysis dilutes the risk of bias by weighting more precise evidence, providing the highest quality evidence to date. Further research is essential to understand teprotumumab’s long-term efficacy and comparative advantages over surgical options. These findings have significant implications for treating persistent proptosis in patients with long-duration TED, potentially offering a non-surgical alternative where options were previously limited.

This systematic review and meta-analysis investigate the efficacy of teprotumumab, a novel monoclonal antibody, in reducing proptosis for patients with long-duration thyroid eye disease (TED). While teprotumumab has shown promise in treating active TED, its effectiveness in chronic cases remained less certain. By synthesizing data from nine individual studies, this meta-analysis aimed to quantify the expected proptosis reduction and evaluate the quality of existing evidence for teprotumumab use in long-duration TED. This research addresses a critical gap in current treatment options, as persistent proptosis in chronic TED has historically been managed primarily through invasive surgical interventions. Understanding teprotumumab’s potential in this context could significantly impact treatment strategies for patients with enduring proptosis, potentially offering a non-surgical alternative for symptom management.

TED, also known as Graves’ ophthalmopathy or Graves’ orbitopathy, is an autoimmune condition characterized by inflammation and fibrosis of the orbital soft tissues. Approximately 40% of patients with Graves’ disease will develop orbital involvement.^1^ At presentation, patients with TED may experience pain, diplopia, tearing, redness, decreased vision, dry eye, eyelid retraction, proptosis, lagophthalmos, extraocular dysmotility, inflammation of the periorbita and conjunctiva and signs of optic nerve compromise. The impact of TED extends beyond physical manifestations, affecting patients’ psychosocial well-being due to disfigurement and functional impairments.^2^ Historically, TED was thought to involve two phases: an initial inflammatory phase followed by a quiescent, fibrotic ‘chronic’ phase. Yet, contemporary findings propose that specific cellular-l evel disease processes endure throughout the prolonged or ‘chronic’ phase, challenging the characterization of this phase as truly ‘inactive’. This was confirmed by Ugradar et al., who demonstrated persistently increased expression of insulin-l ike growth factor-1 (IGF-1) receptors on orbital fibroblasts, making them susceptible to pathological overactivation and fibrogenesis.^3^ This may explain the persistence of proptosis in long-duration TED. In addition, some patients experience recurrent flares of inflammatory disease activity even after reaching the ‘inactive’ phase.^4^ Disease activity is typically monitored using a standardized grading scale based on active TED symptoms called the Clinical Activity Scale (CAS).

The pathogenesis of TED involves the overexpression of the IGF-1 receptor and the thyrotropin receptor (TSH-R), which together form a functional and physical complex on the cell membranes of orbital fibroblasts, B cells and T cells.^5,6^ In Graves’ orbitopathy, current evidence suggests that autoantibodies (anti-TSH-R and, in rare cases, anti-I GF-1R antibodies) bind to the IGF-1R and TSH-R complex, initiating a signalling cascade that leads to the increased production of pro-i nflammatory cytokines, including interleukin (IL)-2, tumour necrosis factor-alpha, and IL-8 by T cells and monocytes.^6–9^ Studies have shown that pathogenic activation of either receptor leads to increased IGF-1R activity, implying the presence of receptor transactivation and IGF-1 dependence in this complex.^10,11^ The downstream effects are mediated by the Akt pathway, which further promotes cell growth and proliferation and inhibits programmed cell death.^12^ Activation of this complex can stimulate hyaluronan production by orbital fibroblasts (OFs), which induces tissue expansion within the orbit and significantly contributes to proptosis.^13^ Typical orbital radiographic findings in TED-related proptosis include orbital fat expansion, enlargement of the extraocular muscles and expansion of the bony orbital cavity, which can persist into the quiescent phase.^14,15^ Failure to address orbital inflammation and proptosis can result in the development of optic nerve compression and the subsequent dysthyroid optic neuropathy (DON). This rare yet severe condition can lead to permanent vision loss.^16,17^ The pathophysiology of disease recurrence and the nature of fibrotic orbital tissue in TED remain incompletely understood. Yet, new findings suggest that the fibrotic phase of TED exhibits cellular-l evel disease activity even in the absence of clinical manifestations.^18^ Understanding this pathogenesis is crucial, as it provides insight into why teprotumumab, which targets the IGF-1 receptor, may be effective even in long-duration cases of TED.

Conventional management strategies for the active phase of TED have predominantly involved immune suppression using agents such as corticosteroids. Although these treatments can reduce inflammatory signs, they have not demonstrated significant efficacy in modifying proptosis.^19,20^ Immunosuppressive agents such as rituximab and corticosteroids are helpful for symptom management in the acute phase of Graves’ disease, but have not been demonstrated to affect proptosis significantly.^21–23^ In the fibrotic phase of TED, debulking and decompressing surgical procedures are the mainstay of treatment for patients with persistent proptosis or vision-threatening compressive optic neuropathy. While these procedures are relatively safe, have low rates of complication rates and are highly efficacious at reducing proptosis, as with all orbital surgeries, they are more invasive and carry risks for complications, including bleeding, infection, vision changes and strabismus.^24,25^

Teprotumumab, a fully human monoclonal immunoglobulin, specifically binds to and blocks the signal transduction of IGF-1R within the IGF-1R– TSH-R complex on OFs and immune cells. This reduces inflammation, extracellular matrix production and soft-tissue remodelling. In January 2020, teprotumumab received approval from the US Food and Drug Administration (FDA) to treat acute TED. The approval was based on phase II and phase III, randomized, double-masked trials (A Multicenter, Double-Masked, Placebo-Controlled, Efficacy And Safety Study Of RV 001, An Insulin-Like Growth Factor-1 Receptor [IGF-1R] Antagonist Antibody [Fully Human], Administered Every 3 Weeks [q3W] By Intravenous [IV] Infusion in Patients Suffering From Active Thyroid Eye Disease [TED]; ClinicalTrials.gov identifier: NCT01868997 and A Phase 3, Randomized, Double-Masked, Placebo-Controlled, Parallel-Group, Multicenter Study Evaluating Teprotumumab [HZN-001] Treatment in Subjects With Active Thyroid Eye Disease; Clinical Trials. gov identifier: NCT03298867) in which teprotumumab outperformed placebo in reducing proptosis in patients who are treatment naive with active TED of fewer than 9-month duration.^26,27^ Orbital imaging conducted throughout the phase III trial and the follow-up OPTIC-X study (Treatment of Graves’ Orbitopathy to Reduce Proptosis With Teprotumumab Infusions in an Open-Label Clinical Extension Study; ClinicalTrials.gov identifier: NCT03461211) revealed a notable reduction in extraocular muscle size and orbital fat volume.^27,28^ Since the approval by the US FDA, substantial evidence has been published confirming the ability of teprotumumab treatment to effectively reduce proptosis in active, inflammatory TED.^29^ However, similar efficacy has not yet been confirmed to the same extent for treating persistent proptosis in long-duration, inactive and non-i nflammatory disease states.

Recently, attention has shifted towards the ability of teprotumumabs to treat persistent symptoms in cases of prolonged TED. Given the sustained overexpression of IGF-1R on local orbital cells in chronic TED, it is plausible that teprotumumab could improve proptosis even after many years of clinical stability. In July 2023, Horizon Therapeutics (West Palm Beach, FL, USA) disclosed findings from its first phase IV clinical trial (Efficacy and Safety of Teprotumumab in Patients With Thyroid Eye Disease of Long Duration and Low Disease Activity; ClinicalTrials.gov identifier: NCT04583735), which confirmed teprotumumab’s capacity to significantly diminish proptosis in cases of long-duration TED.^30^ Additional early reports from other researchers similarly suggest that teprotumumab may effectively reduce proptosis in such cases.^31–34^

While many reports and studies appear to confirm the efficacy of teprotumumab in long-duration TED, current data from studies are constrained by small sample sizes, risks of biases and between-study variabilities regarding the average reductions in proptosis. This systematic literature review aims to thoroughly explore all case series and clinical trials documenting the extent of teprotumumab’s impact on proptosis in long-duration TED (defined as a time from diagnosis of more than 9 months) and to further document the strengths, limitations and risks of bias present in each study. Long-duration TED is consistently defined in the literature as a condition persisting for more than 9 months after the initial onset of symptoms.^26,27,31–33^ The 9-month threshold is an approximate demarcation between TED’s acute and chronic phases. While this provides researchers and clinicians with a standardized time frame for classification and treatment approaches, the actual clinical distinction may depend more on individual CAS scores and current symptom presentation. For the practical purposes of this review, the 9-month time frame was used to avoid erroneous inclusion of participants with recent-onset TED. Subsequently, a meta-analysis will be performed on the aggregated data to ascertain the anticipated degree of proptosis reduction that can be expected in these patients following teprotumumab treatment. Consequently, this approach will yield greater statistical power and generalizability than any individual study alone. In addition, our meta-analysis will be the first to incorporate newly published data from the recent Horizon phase IV trials.^30^ We present this article in accordance with the Preferred Reporting Items for Systematic Reviews and Meta-Analyses (PRISMA) reporting checklist.

## Methods

The full systematic review was conducted in accordance with PRISMA guidelines. The review protocol is registered on INPLASY (ID: 202380051; Protocol 5157), and the data are published and publicly available on SRDR+ (ID: 5101). To identify relevant studies, the search strategy involved querying five databases: Cochrane, Science Direct, PubMed, Google Scholar and Embase. All studies in which the abstract or title included ‘teprotumumab’ and ‘chronic thyroid eye disease’, ‘Graves ophthalmopathy’ or ‘thyroid-associated orbitopathy’ were examined. Synonyms for ‘chronic’, such as ‘long-duration’, ‘long-standing’ and ‘advanced’, were also tested to ensure comprehensive coverage of relevant literature. Studies eligible for synthesis met the following inclusion criteria: clinical trials, case series, retrospective or prospective studies, and participants with chronic TED defined by duration over 9 months from symptom onset, multiple participants completing treatment regimens and objective outcome measures were recorded at the start and after the final infusion. While the full course is eight infusions, our analysis included patients who received at least seven infusions, as this approach reflects common clinical practice and still provides valuable insights into teprotumumab’s effects. The primary outcome of interest is the reduction in proptosis (measured in millimetres). Results and relevant data were extracted by a single reviewer and verified and cross-checked by a second researcher.

**Figure 1:**
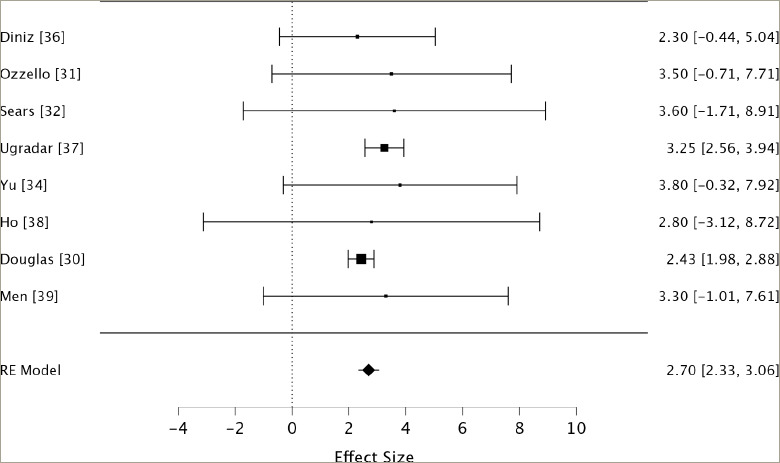
Forest plot^30–32,34–38^

*This forest plot shows the mean proptosis reduction and 95% CIs for eight individual studies and the RE model. Effect sizes are in millimetres. The size of each square is proportional to the study’s weight in the meta-analysis. The diamond represents the overall effect size, with its width indicating the 95% CI. The RE model shows a pooled estimate of 2.70 mm proptosis reduction (95% CI: 2.33–3.06 mm) across 172 analysed orbits.*

*RE = random effects; CI = confidence interval.*

**Figure 2:**
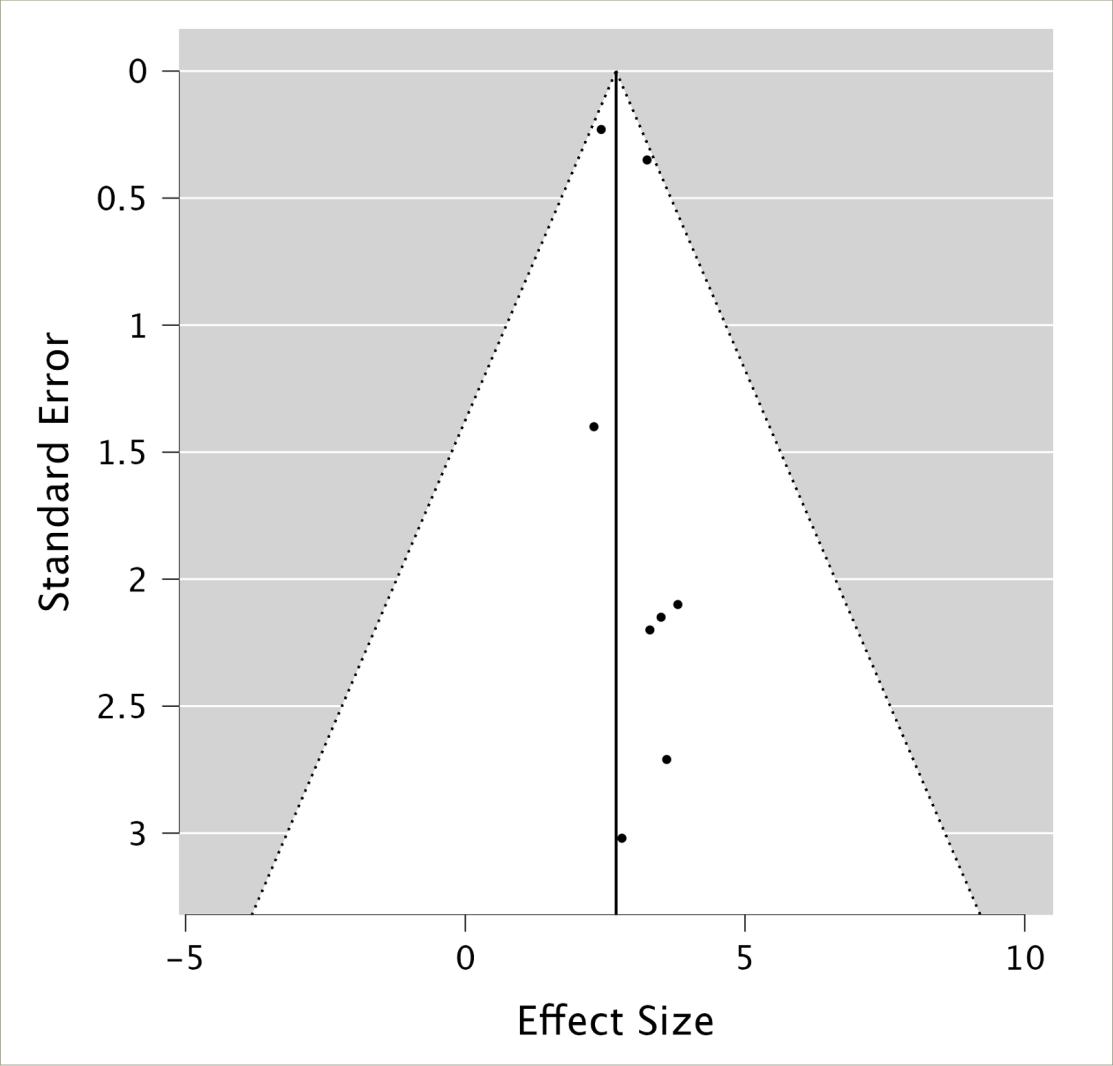
Funnel plot

The measures of proptosis reduction include weighted and cumulative averages. Data weighting was determined using the inverse variance method (inverse square of standard deviation) and random-effects model. Cumulative means were generated based on the sample sizes of included studies. Standard error and 95% confidence intervals were calculated using the cumulative and weighted averages to determine the precision of the estimates. Heterogeneity analysis was performed using Cochran’s Q statistic. JASP software (copyright 2013–2024 University of Amsterdam) was used to run a weighted meta-analysis using the random-effects model (Figure 1), which considers within-study and estimated between-study variance, and accounts for potential heterogeneity.^30–32,34–38^ Results from this analysis are displayed in a forest plot (Figure 1) and funnel plot (Figure 2). The Grading of Recommendations Assessment, Development and Evaluation approach was used to evaluate the certainty of evidence, and the Risk Of Bias In Non-randomized Studies of Interventions (ROBINS-I ) criterion was used to aid the assessment of bias.^39^ Sensitivity analysis was performed by sequentially removing each study from the weighted synthesis and recalculating the weighted means and standard deviations. The quality of evidence was assessed in terms of sample size, study design, precision of measurements, presence of long-term follow-up and reporting of adverse events. The risk of bias was evaluated based on criteria, including the presence of a control group, pre-treatment CAS ≤1 among participants, consistency in prior treatment histories among participants and standardized treatment administration with particular attention to the number of teprotumumab infusions administered.

## Results

**Figure 3:**
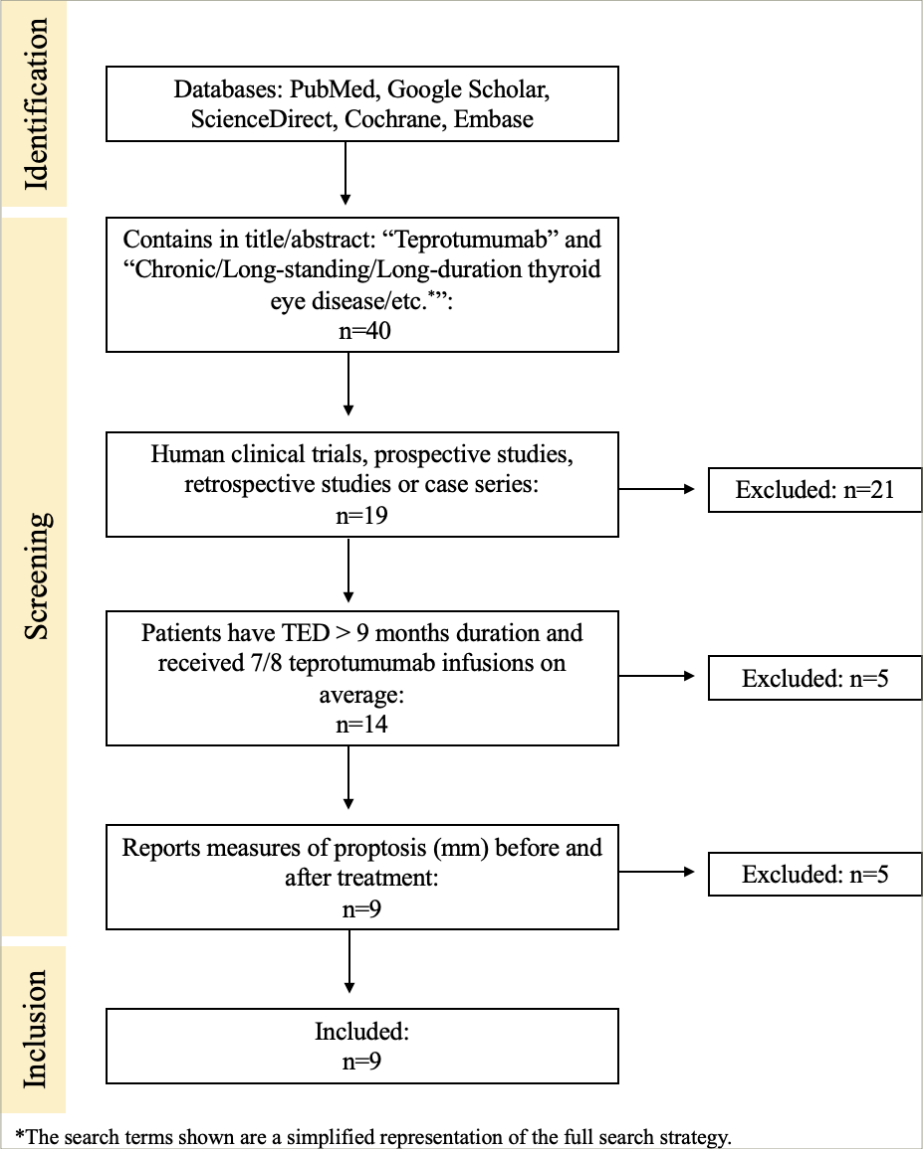
Diagram of the search strategy and results

Forty publications met the advanced search criteria and were reviewed for inclusion criteria; ultimately, nine studies were included in the meta-analysis. Twenty-one papers were excluded because they were reviews or narratives that did not present primary evidence. Five publications were excluded because their primary outcome measures did not include proptosis reduction. Five articles were excluded because their studies included participants with active or acute TED and did not provide separate data for those participants with inactive or long-duration TED (Figure 3). The evaluation of evidence quality and risk of bias is displayed in *Table 1*, and the extracted data from each included study are shown in *Table 2*.^30–38^

Diniz et al. presents a cross-sectional cohort study investigating teprotumumab treatment in 21 patients with a heterogeneous history of TED.^35^ The provided data differentiate between patients with acute TED, who were excluded from our meta-analysis, and those with longer-duration, ‘stable’ TED, whose data were extracted and included. In this study, 11 patients with long-duration TED demonstrated a mean proptosis reduction of 2.3 ± 1.4 mm (p<0.001) (*Table 2*). Data were recorded only on the more proptotic eye in each participant. Limitations of this study include a variable number of teprotumumab infusions among patients, an unblinded design, lack of placebo control, variable pre-treatment CAS, unavailable long-term follow-up and heterogeneous treatment histories among participants. Strengths of this study include prospective design, reporting of individual patient data, reporting of side effects and analysis of multiple primary and secondary outcomes in addition to proptosis reduction. The authors found that teprotumumab was also significantly efficacious in improving extraocular motility, but it did not impact strabismus or margin-to-reflex distance in patients with long-duration TED. The strength of evidence is moderate, and the risk of bias is also moderate.^35^

*The funnel plot of the meta-analysis, where each point represents an individual study, with the x-axis showing the effect size (proptosis reduction in millimetres) and the y-axis showing the standard error of the effect size estimate. The vertical line represents the pooled effect estimate from the random-effects model. Marked asymmetry in the plot may be indicative of publication bias.*

Ozzello et al. conducted a retrospective case series, including patients with TED of over 9 months of disease duration and CAS of <1.^31^ These authors report a mean proptosis reduction of 3.5 ± 2.15 mm (p=0.002) among the 18 study orbits from nine participants (*Table 2*). Regarding side effects, the authors reported that three patients experienced infusion myalgia, two experienced hair thinning, one experienced tinnitus and one developed hyperglycaemia. Limitations of this study include its retrospective design, inclusion of participants with heterogeneous treatment histories, unavailable long-term follow-up, low precision of measurements and small sample size. Strengths of the study include measurements taken from both eyes or orbits, completion of the entire infusion series by all participants, inclusion criteria of pre-treatment CAS <1 and specific side effects reported. Overall, the quality of evidence from this paper is low, and the risk of bias is moderate.^31^

Sears et al. conducted a prospective, longitudinal case study analysing teprotumumab efficacy in treating TED in patients with DON who were not candidates for surgical decompression.^32^ The authors reported a mean proptosis reduction of 3.6 ± 2.71 mm (p<0.0001) among 14 orbits included in their analysis (*Table 2*). The article reported individual outcomes for 10 patients; 7 with long-duration TED, which were included in our meta-analysis, and 3 with acute TED, which were excluded. Results were reported for both orbits. The authors noted that 50% of participants received orbital magnetic resonance imaging or computed tomography (CT) scans, which confirmed improvement in orbital crowding. Importantly, all patients who presented with relative afferent pupillary defects (RAPD) or decreased colour vision experienced resolution of RAPD or improvement in colour vision following teprotumumab treatment. The study did not report side effects. Limitations of this study include participants with heterogeneous treatment histories, unblinded design, lack of placebo control, variable pre-treatment CAS among participants, unavailable long-term follow-up, low precision of recorded proptosis measurements and a small sample size. Placebo control was impossible for this study, as participants had DON, which threatened permanent vision loss without treatment. Strengths of this study include its prospective design, the fact that all participants completed all eight teprotumumab infusions, and its unique finding that teprotumumab can aid in treating DON caused by TED. Overall, this evidence quality of this paper is low, and the risk of bias is moderate.^32^

*Schematic of search strategy. ‘n’ refers to the number of publications screened at each stage.*

*TED = thyroid eye disease.*

**Table 1:** Evaluation of evidence quality and risk of bias^30–38^

Study	Design	Precise?	Long-term follow-up?	AEs reported?	Control?	Initial CAS ≤1?	Homogeneous treatment histories?	All participants received 8/8 infusions?
Diniz et al. (2021)^35^	Prospective			✓				
Ozzello et al. (2022)^31^	Retrospective			✓		✓		✓
Sears et al. (2021)^32^	Prospective							✓
Ugradar et al. (2022)^36^	Retrospective	✓		✓				
Vinson and Kirzhner (2022)^33^	Retrospective			✓				✓
Yu et al. (2022)^34^	Prospective							
Ho et al. (2023)^37^	Prospective			✓		✓		
Douglas et al. (2023)^30^	Prospective	✓		✓	✓	✓	✓	✓
Men et al. (2024)^38^	Retrospective		✓	✓		✓		

*Criteria and results for evaluating evidence strength and risk of biases among nine included studies. A study was determined to be precise if the standard deviation of the mean proptosis reduction reported was less than 15% of the measurement value. Long-term follow-up was defined as having a duration greater than 6 months following completion of treatment. Initial CAS refers to the Clinical Activity Scale score of participants before beginning treatment with teprotumumab. The criterion of homogeneous treatment histories of participants is met if treatment history among included patients was consistent, especially concerning orbital decompression surgery.*

*AEs = adverse effects; CAS = Clinical Activity Scale; Control = control group.*

Ugradar et al. conducted a retrospective, longitudinal study involving 35 patients with long-duration TED.^36^ The researchers reported a mean proptosis reduction of 3.25 ± 0.35 mm (p<0.01) following treatment with teprotumumab (*Table 2*). They used manual segmentation of CT scans to determine the volume of orbital muscles and fat before and after treatment. Participants who had previously received rituximab treatment were excluded. The study also reported a mean orbital muscle volume reduction of 2011 ± 1847 mm^3^ for the study eye and 1620 ± 1759 mm^3^ for the fellow eye. The study did not report specific side effects from teprotumumab infusions, but did note that no side effects were severe enough to cause participants to discontinue treatment. Limitations of this study include its retrospective and unblinded design, lack of placebo control, inclusion of participants with heterogeneous treatment histories, variable pre-treatment CAS among participants, unavailable long-term follow-up and inconsistent number of teprotumumab infusions. Strengths of this study included high precision of proptosis measurements, exclusion of patients with prior treatment from rituximab or tocilizumab, data recorded from both eyes and evaluation of several outcome measures. Overall, the quality of evidence from this paper is high, and the risk of bias is also potentially high.^36^

Vinson et al. presented a retrospective case series involving five patients with TED of greater than 9 months of duration.^33^ Here, data from both eyes of each participant revealed a mean proptosis reduction of 3.4 mm (*Table 2*) across the 10 orbits analysed. Standard error of measurements was not provided; thus, this value was not included in the weighted meta-analysis. Pre-treatment CAS was not reported. The study reported specific side effects, including three patients with leg cramping, one with stomach cramping, one with worsened hearing loss, one with brittle nails, one with loss of taste and one with mild hair loss. Two patients did not experience any side effects. Limitations of this study include its retrospective design, lack of control, heterogeneous treatment history among participants, variable pre-treatment CAS, low sample size, unavailable long-term follow-up and lack of reported standard error. Strengths of the study include detailed reporting of side effects and ensuring all included participants received eight teprotumumab infusions. Overall, the evidence quality of this study is low, and the risk of bias is moderate.^33^

Yu et al. conducted a prospective case series that investigated the effects of teprotumumab in six patients with chronic, clinically active TED.^34^ The authors found a mean proptosis reduction of 3.8 ± 2.1 mm among five included patients (*Table 2*). Side effects were not reported. Five participants from this study were included in our analysis, as one participant in this study had only received six of eight infusions. Limitations of this study include its unblinded design, lack of placebo control, small sample size, unavailable long-term follow-up, participants with heterogeneous treatment histories, low precision of measurements, unequal number of teprotumumab infusions, no reporting of adverse events and variable pre-treatment CAS. Strengths include a prospective design and a participant pool with an average TED duration of 125 months, the longest across the studies analysed. Overall, this paper’s evidence quality is low, and the risk of bias is high.^34^

**Table 2:** Results of systematic review^30–38^

Study	Participants	Orbits analysed	Mean proptosis reduction (mm)	p-value	Quality of evidence	Risk of bias
Diniz et al. (2021)^35^	11	11	2.30 ± 1.40	<0.001	Moderate	Moderate
Ozzello et al. (2022)^31^	9	18	3.50 ± 2.15	0.002	Low	Moderate
Sears et al. (2021)^32^	7	14	3.60 ± 2.71	<0.0001	Low	Moderate
Ugradar et al. (2022)^36^	31	62	3.25 ± 0.35	<0.01	High	High
Vinson and Kirzhner (2022)^33^	5	10	3.40	NR	Low	Moderate
Yu et al. (2022)^34^	5	5	3.80 ± 2.10	NR	Low	High
Ho et al. (2023)^37^	12	12	2.80 ± 3.02	<0.01	Moderate	Moderate
Douglas et al. (2023)^30^	41	41	2.43 ± 0.23	0.0004	Very high	Low
Men et al. (2024)^38^	8	8	3.30 ± 2.20	0.003	Moderate	Moderate

*This table highlights the data extracted from each included study and summarizes the quality of evidence and risk of bias present. ‘Participants’ indicates the number of individuals in each study. ‘Orbits analysed’ shows the total number of eyes included in the analysis. When these numbers are equal, only one eye per participant was studied. When 'Orbits analysed' is double ‘participants’, both eyes of each participant were included in the analysis. If reported by the original authors, the standard error of measurements was included in the table.*

*NR = not reported.*

Ho et al. conducted an observational cross-sectional cohort study of 71 patients from 15 institutions with active or inactive TED treated with teprotumumab.^37^ Of these 71 patients, 12 were defined as having minimal-to-l ow activity TED over 9 months. Data were exclusively extracted from these 12 patients for the meta-analysis. Among these patients, they found a mean proptosis reduction of 2.80 ± 3.02 mm (*Table 2*). Patients with active TED comparatively experience a mean reduction in proptosis of 3.2 ± 5.08 mm (p<0.01). This study had the lowest precision of measurements among the included papers. The authors also reported that teprotumumab treatment significantly improved CAS in patients with stable TED (p =0.05), but did not significantly improve extraocular range of motion or margin-to-distance reflex. The paper noted adverse events, including muscle cramps, hair loss, hyperglycaemia, hearing changes, fatigue and gastrointestinal upset. However, the distribution of these side effects between active and stable TED patients is unclear. Strengths of this paper include its prospective design, measurement of several outcome measures, minimal pre-treatment CAS, reporting of adverse effects and its ability to compare the effects of teprotumumab in active versus stable TED. Limitations of this paper include low precision of measurements, unblinded design, lack of placebo control, heterogeneous patient treatment histories, lack of long-term follow-up and unequal number of teprotumumab infusions between participants. Overall, the evidence quality of this study is moderate, and the risk of bias is also moderate.^37^

Douglas et al. recently published preliminary results from the phase IV Horizon Therapeutics double-blind, placebo-controlled trial.^30^ Here, the researchers reported a mean proptosis reduction of 2.43 ± 0.23 mm among 41 included patients (*Table 2*), compared with 0.91 ± 0.32 (p =0.004) among controls. This study provided the highest degree of measurement precision and thus was heavily weighted in the meta-analysis. The researchers defined inclusion criteria as 2–10 years of TED symptoms with stability for at least 1 year and a corresponding CAS score of 0 or 1. Confounding variables were controlled through stringent inclusion criteria to exclude patients with heterogeneous treatment histories or active TED flares that would otherwise confound data. Participant selection was performed with meticulous attention to the risk of bias, as this study used a multi-centre, placebo-controlled, double-masked, parallel-group trial. The reported side effects included hyperglycaemia, hearing loss and infusion reactions, although no significant difference in adverse events was observed in the placebo group. All participants completed the full series of infusions. Although the generalizability of the trial is somewhat limited due to its sample size, it exhibits greater generalizability than other publications in this analysis, thanks to its comparatively larger sample size and high precision of measurements. One limitation of this study is the lack of long-term follow-up. This is the most well-designed, reliable and significant study on proptosis reduction in long-duration TED. The strength of evidence in this paper is very high, and the risk of bias is low.^30^

Men et al. published results from a multi-centre, retrospective study of patients with moderate-to-severe TED who were treated with teprotumumab after failing conventional therapies.^38^ Inclusion criteria were defined as patients who experienced incomplete responses to prior therapy with persistence or reactivation of disease with proptosis, diplopia or orbital inflammatory symptoms. While the study includes 66 participants, only 8 could be classified as having long-duration TED and were included in our analysis. Nonetheless, it is significant to note that the authors found that, among 66 patients whose disease failed to respond to prior therapies, including corticosteroids and orbital decompression surgery, teprotumumab treatment led to a significant mean proptosis reduction of 3.1 ± 2.4 mm (p<0.001). The study provides data on eight participants included in the study with clinically inactive, long-duration TED, who achieved a mean proptosis reduction of 3.3 ± 2.2 mm (p=0.003), and these data were included in our analysis. Adverse events reported included muscle spasms, hearing impairment, alopecia, fatigue, diarrhoea, hyperglycaemia and menstrual irregularities. They also reported a significant decrease in proptosis, even in patients with TED who had previously undergone orbital decompression surgery. Strengths of this paper include subgroup analysis based on disease duration, clinical activity and treatment history, precise inclusion criteria, reporting of adverse events and relatively long-term follow-up with a mean follow-up duration of 37 weeks. Limitations include only eight participants with long-duration TED, low precision of proptosis measurements, retrospective design, heterogeneous treatment histories, lack of placebo control and not all participants completing eight infusions. Overall, the evidence quality of this study is moderate, and the risk of bias is also moderate.^38^

Overall, the studies consistently demonstrated a significant reduction in proptosis following teprotumumab treatment in patients with long-duration TED, with mean reductions ranging from 2.30 to 3.80 mm across the included studies. The p-values across studies ranged from <0.0001 to 0.01, indicating strong statistical significance in the observed proptosis reductions.

## Data analysis

Cumulative means, weighted by sample size, and weighted means, weighted by precision, were both generated, as the relative paucity of data available may skew one or the other. Data from all included studies were pooled into a cumulative meta-analysis. Data from the eight studies that provided standard measurement error were factored into weighted meta-analyses. Precision-based weighting was assigned according to the inverse variance and random effect models. In addition, studies that reported mean proptosis reduction values for both the left and right eye were grouped and averaged to allow for equivalent comparison across studies and avoid misrepresenting left and right eye results as separate trials. Therefore, for proptosis measurements, data by Ugradar et al. will be n=62 and mean =3.25 ± 0.35 mm, and the data by Ozzello et al. will be n=18 and mean =3.5 ± 2.15 mm.

## Calculations

### Cumulative meta-analysis

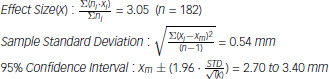



### Heterogeneity analysis

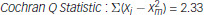



**Table 3:** Results of meta-analysis

Meta-analysis	N (orbits)	Mean proptosis reduction (mm)	95% Confidence interval (mm)
Cumulative	182	3.05 ± 0.54	2.70 to 3.40
Weighted	172	2.69 ± 0.53	2.61 to 2.77
RE Model	172	2.70	2.33 to 3.06

*This table displays the results of the two meta-analyses performed in this review. The first involved a cumulative analysis, pooling data from all nine studies according to sample sizes. The second involved a weighted analysis whereby only data from the eight studies that reported standard error could be included. Precision-based weighting was assigned to study data based on the inverse variance method (inverse of squared standard deviation). The RE model used a weighted synthesis according to between-study and within-study heterogeneity.*

*RE = random effects.*

### Weighted meta-analysis

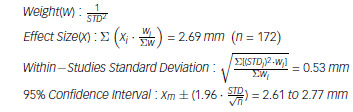



### Weighted heterogeneity analysis

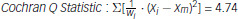



## Discussion

Nine studies met the inclusion criteria of the systematic search. The results of the cumulative meta-analysis (weighting by sample size) show a mean proptosis reduction of 3.05 ± 0.54 mm, and the results of the weighted meta-analysis (weighting by precision) show a mean proptosis reduction of 2.69 ± 0.53 mm. In patients with long-duration TED included in this meta-analysis, the 95% confidence interval for the cumulative analysis reveals an expected proptosis reduction between 2.70 and 3.40 mm, and the weighted analysis reveals an expected proptosis reduction between 2.61 and 2.77 mm (*Table 3*). Meta-analysis using the random-effects (RE) model found a mean effect size of 2.70 mm with a 95% confidence interval from 2.33 to 3.06 mm (*Table 3*).

The high precision of studies by Ugradar et al. and Douglas et al. strongly draws the weighted mean towards the values reported by these authors. This is reflected in the sensitivity analysis (*Table 4*).^30–32,34–38^ Comparatively, the results of this study showed a standard deviation >2.00 mm, which were relatively inconsequential in the weighted meta-a nalysis. Still, they were influential in the cumulative meta-analysis based on their sample sizes. Due to the lower risk of bias in the higher precision studies, the weighted means and weighted confidence intervals may provide a more accurate estimation. The often large standard errors of measurements in several included studies highlight an important source of bias throughout included papers: measurement bias. Proptosis measurements across all the included studies involved manual procedures conducted by one or more researchers employing an exophthalmometer. This approach inherently introduces the potential for procedural bias due to human involvement, even when conducted by the same researcher, adding to the inherent bias associated with the measuring instrument.

**Table 4:** Sensitivity analysis^30–32,34–38^

Study removed	New weighted mean
Diniz et al. (2021)^35^	2.70 ± 0.50 mm
Ozzello et al. (2022)^31^	2.69 ± 0.49 mm
Sears et al. (2021)^32^	2.69 ± 0.49 mm
Ugradar et al. (2022)^36^	2.47 ± 0.59 mm
Yu et al. (2022)^34^	2.69 ± 0.50 mm
Ho et al. (2023)^37^	2.69 ± 0.50 mm
Douglas et al. (2023)^30^	3.21 ± 0.85 mm
Men et al. (2024)^38^	2.69 ± 0.50 mm

*This table displays the sensitivity analysis results, which were performed following the same statistical procedure as the primary analysis. This involved selectively removing each individual study and recalculating the weighted means and standard deviations, illustrating the sensitivity of each contributing study’s data.*

The homogeneity of the included studies was established through multiple statistical analyses. Both cumulative and weighted meta-analyses yielded Q values (2.33 and 4.74, respectively) smaller than their corresponding degrees of freedom (9 and 8, respectively), indicating that study variation is attributable to random chance rather than significant heterogeneity.^40^ While some asymmetry was observed, suggesting the possibility of mild publication bias, the overall distribution of studies does not indicate severe skewing of results. The funnel plot (Figure 2) shows a range of study precisions with no extreme outliers. However, the slight asymmetry and potential gap in the lower left corner suggest that smaller studies with less significant results might be underrepresented. Given this meta-analysis’s relatively small number of studies, these observations should be interpreted cautiously. Nonetheless, the funnel plot analysis supports the overall robustness of our findings while acknowledging the potential for some degree of publication bias, which may result in overestimation of the effect of teprotumumab on proptosis reduction.

The included studies were predominantly well-designed, though many were open to bias. Two large sources of bias across included studies are heterogeneous treatment histories and variable pre-treatment CAS. Regarding heterogeneous treatment histories, it is unknown how prior histories of various other Graves’ disease treatments may affect teprotumumab’s ability to reduce proptosis. Recent evidence demonstrates that teprotumumab treatment can significantly reduce proptosis (by more than 2.0 mm) in many patients with steroid-resistant TED.^41^ This finding further highlights its potential effectiveness across various TED presentations, including patients with diverse treatment histories, such as corticosteroid use. Regarding variable pre-treatment CAS, several of the included studies defined long-duration TED as the duration from symptom onset over 9 months, regardless of pre-treatment CAS. In contrast, others specified that a pre-treatment CAS of 1 or less was required to meet the definition of long-duration TED. This highlights the need for a universal definition of long-duration TED to provide future investigators with standardized inclusion criteria for investigating the different disease stages. As a result, studies that included patients with higher pre-treatment CAS introduce the possibility that certain participants in our meta-analysis may have been experiencing ‘flare-ups’ of acute symptoms. Alternatively, elevated CAS in the second phase of TED may be more likely to result from persistent orbital congestion rather than active inflammation. Still, more research is required to elucidate the interaction between CAS and teprotumumab’s ability to reduce proptosis, as it is not known whether proptosis in patients with elevated pre-treatment CAS responds more significantly to teprotumumab treatment. Despite this variability in inclusion criteria, the reported proptosis reductions were similar across the studies analysed.

Notably, the recent data from the phase IV Horizon trial, unique for its double-masked, placebo-controlled design and relatively larger participant pool, demonstrates a slightly smaller reduction in proptosis (2.43 mm) compared with the other studies analysed.^30^ This study exhibited the highest precision of measurements, the most rigorous inclusion criteria and the strongest control over potentially confounding variables. Thus, this trial provided the highest overall evidence quality, with the lowest risk of bias. The trial’s stringent inclusion criteria excluded patients with a CAS greater than 1, recent symptom developments (within the past year), or prior treatments involving irradiation, orbital decompression surgery or strabismus surgery. The data reported in this study, which aligns closely with our weighted meta-analysis, may provide a more accurate representation of the actual effect size, due to its rigorous methodology and larger sample size. The absence of clinical or inflammatory disease activity in these participants suggests a possible correlation between the state of disease activity and teprotumumab’s impact on proptosis. This implies that individuals with lower clinical disease activity might experience slightly smaller reductions in proptosis. However, further investigation is necessary to substantiate this potential association.

The average proptosis reductions identified in this meta-analysis are marginally less than those observed in patients with acute inflammatory TED during the Horizon phase III clinical trial.^26^ Within this trial, 41 patients with acute TED experienced a mean proptosis reduction of 3.32 mm.^26^ Hence, individuals with inactive TED experiencing ongoing proptosis may anticipate a comparable or slightly less pronounced decrease in proptosis when treated with teprotumumab compared with those with active TED. These findings imply that metabolic regulation of local fibroblasts of the orbital tissue is similar in acute and long-duration TED. The enduring presence of the TSH-I GF-1 receptor complex suggests that OFs in long-duration TED continue to be vulnerable to activation by Graves’ disease antibodies. Therefore, inhibition of these cells by teprotumumab would prevent stimulation of the Akt pathway, enabling programmed cell death and appropriate cellular and metabolic turnover. This could explain the significant and consistent proptosis reductions observed in active and inactive TED. In addition, other mechanisms yet to be elucidated may be at play, by which teprotumumab further decreases inflammation, decreases orbital tissue production and increases tissue turnover. More research into pathophysiology is required to shed light on these cellular effects.

Individually, many of these studies cannot conclusively address the extent to which teprotumumab treatment significantly reduces proptosis in patients with long-duration disease, primarily because of intrinsic limitations within each study. However, this meta-analysis addresses these by amalgamating data among case series, cohort studies and clinical trials to enhance statistical power, mitigate individual biases, and dilute the influence of confounding variables. This is further achieved by weighting studies with relatively greater measurement precision. Therefore, this analysis provides the most compelling evidence of the degree of proptosis reduction that can be anticipated with teprotumumab treatment in late-stage TED. The broader applicability of the conclusions drawn from this meta-analysis surpasses that of any individual study in isolation.

The review is limited by the lack of long-term follow-up in the included case series and trials, leaving questions about the longevity of the proptosis reductions observed with teprotumumab. Consequently, the proptosis recurrence rate over subsequent years post-treatment remains uncertain. More extended longitudinal studies are needed to investigate teprotumumab’s lasting effectiveness by monitoring for proptosis recurrence. This aspect is relevant when comparing efficacy of teprotumumab, long-term advantages and risks versus orbital decompression surgery. Prospective, randomized controlled trials comparing teprotumumab with surgical interventions in long-duration TED would provide valuable comparative efficacy data. Additional studies are needed to elucidate how variable pre-treatment CAS and diverse treatment histories might influence the efficacy of teprotumumab in treating proptosis in long-duration TED.

Overall, this meta-analysis quantifies a promising advancement in the pharmaceutical treatment of enduring proptosis in TED, with important clinical implications. The consistent reduction in proptosis observed across studies suggests that teprotumumab could offer a valuable non-surgical treatment option for patients with long-duration TED who continue to experience proptosis. This could potentially reduce the need for invasive orbital decompression surgeries in some patients, altering the current treatment paradigm for chronic TED.
